# Approaches for community intervention and research priority setting to reduce health inequalities: a scoping review

**DOI:** 10.1093/pubmed/fdaf151

**Published:** 2025-12-04

**Authors:** Catherine E Shuttleworth, Jack M Birch, Lauren Bell, Michael Ogunyemi, Cameron D Ley, Harmony Lully, John Wilcox, Richard Grant, Jane Whitehouse, Naila Dracup, Sophie Staniszewska, Yen-Fu Chen

**Affiliations:** NIHR Health Determinants Research Collaboration Coventry, Coventry City Council, One Friargate, Coventry, CV1 2GN, UK; NIHR Health Determinants Research Collaboration Coventry, Coventry City Council, One Friargate, Coventry, CV1 2GN, UK; NIHR Policy Research Unit Behavioural and Social Sciences, Population Health Sciences Institute, Faculty of Medical Sciences, Baddiley-Clark Building, Richardson Road, Newcastle University, Newcastle upon Tyne, NE2 2AX, UK; NIHR Health Determinants Research Collaboration Coventry, Coventry City Council, One Friargate, Coventry, CV1 2GN, UK; NIHR Health Determinants Research Collaboration Coventry, Coventry City Council, One Friargate, Coventry, CV1 2GN, UK; NIHR Health Determinants Research Collaboration Coventry, Coventry City Council, One Friargate, Coventry, CV1 2GN, UK; NIHR Health Determinants Research Collaboration Coventry, Coventry City Council, One Friargate, Coventry, CV1 2GN, UK; NIHR Health Determinants Research Collaboration Coventry, Coventry City Council, One Friargate, Coventry, CV1 2GN, UK; NIHR Health Determinants Research Collaboration Coventry, Coventry City Council, One Friargate, Coventry, CV1 2GN, UK; NIHR Health Determinants Research Collaboration Coventry, Coventry City Council, One Friargate, Coventry, CV1 2GN, UK; NIHR Health Determinants Research Collaboration Coventry, Coventry City Council, One Friargate, Coventry, CV1 2GN, UK; Centre for Evidence and Implementation Science, School of Social Policy and Society, University of Birmingham, Birmingham, B15 2TT, UK; Warwick Applied Health, Warwick Medical School, University of Warwick, Coventry, CV4 7AL, UK; NIHR Health Determinants Research Collaboration Coventry, Coventry City Council, One Friargate, Coventry, CV1 2GN, UK; Warwick Applied Health, Warwick Medical School, University of Warwick, Coventry, CV4 7AL, UK; NIHR Health Determinants Research Collaboration Coventry, Coventry City Council, One Friargate, Coventry, CV1 2GN, UK; Centre for Evidence and Implementation Science, School of Social Policy and Society, University of Birmingham, Birmingham, B15 2TT, UK; Warwick Applied Health, Warwick Medical School, University of Warwick, Coventry, CV4 7AL, UK

**Keywords:** social determinants, research, communities

## Abstract

**Background:**

There is growing attention on research and intervention prioritization regarding the social determinants of health to address health inequalities. Community involvement in this prioritization is centrally important. This scoping review aimed to identify: (i) examples of priority setting regarding the social determinants of health and (ii) methods for involving local communities in research or intervention prioritization.

**Methods:**

Searches were conducted in Medline, Social Policy & Practice, Applied Social Sciences Index & Abstracts, CINAHL, and Carrot2 in May 2024. Eligible studies reported prioritization with communities for interventions or research about the social determinants of health. Studies reported primary research in high-income countries. A narrative synthesis was undertaken, with a review team involving different professionals and public contributors.

**Results:**

Eighteen studies were included. Community prioritization methods varied, though commonly included participatory approaches, with additional reports of Delphi exercises, a super-setting approach, a nominal group technique, a deliberative exercise using a serious game, and a modified James Lind Alliance process.

**Conclusions:**

Meaningful community involvement in research and intervention prioritization offers critical opportunities to reduce existing health inequalities. Participatory and coproduced approaches are valuable to research collaborations, funders, and public health organizations, which should ensure trust, accessibility, and inclusion to involve diverse and underrepresented communities.

## Introduction

Health inequality is a long-standing social challenge that remains on the policy agenda for many countries, yet there are signs that inequalities have worsened in recent years.^1^^,^^2^ As interventions targeting individuals’ health behaviours tend to produce limited short-term impact, tackling the root causes (i.e. social determinants of health) is critical. Social determinants of health are the conditions that people are born, grow, work, and live in that shape health outcomes, such as housing, education, racism, and employment.^3^ Understanding communities’ concerns regarding these determinants is crucial to ensure research, policies, and interventions are effectively prioritized and relevant.^4^

Involving the public in creating and co-producing solutions to tackle health inequalities can improve democracy, the evidence base, and public acceptance of resulting policies and interventions.^5^ The National Co-production Advisory Group, part of the Think Local Act Personal Partnership, describes a ladder of coproduction, moving through coercion, educating, informing, consultation, engagement, codesign, and coproduction.^6^ Successful co-production brings together communities, professionals, and other groups to design research or interventions, achieving a genuine balance of power throughout the process.^7^ Involvement of diverse groups can minimize omission of relevant priorities, create ownership over priorities, ensure outcomes best address community needs, and reduce duplication of resource.^8^

The importance of collaborative research in tackling health inequalities has been recognized through recent developments and funding of programmes in the UK.^9–11^ The National Institute for Health and Care Research (NIHR) established 30 Health Determinants Research Collaborations (HDRCs) that bring together local authorities, academic institutions, voluntary and community organizations, and local communities to facilitate collaborative research to inform policies and interventions.^12^ HDRCs are place-based collaborations, meaning they involve partners and communities in a defined geographical area, aligned to the host local authority, to target local inequalities and priorities. To ensure meaningful research is undertaken by collaborations like HDRCs, it is critical to understand the needs and priorities of local communities.

Despite a growing body of literature on public involvement and priority setting in health-related research, most reviews have considered clinical research or individual behaviour change interventions.^13–15^ To our knowledge, no published reviews have investigated community priority setting for research and interventions relating to the social determinants of health. This scoping review was conducted by HDRC Coventry^16^ to produce learning that could be implemented locally, by other HDRCs, and similar programmes. Aligned with the collaborative principles of HDRCs, the study team involved researchers, public health practitioners, and two public contributors throughout.^17^ Objectives were to:

- Explore methods for involving or engaging local communities in research or intervention priority setting
- Identify examples of place-based priority setting exercises related to the social determinants of health

## Methods

### Search strategy

Medline, Social Policy & Practice, Applied Social Sciences Index & Abstracts, CINAHL, and Carrot2 were searched by an Information Specialist in May 2024 using indexed terms and keywords related to deprivation, community participation and involvement, health determinants, priority setting, and research methods. Grey literature was searched via Carrot2. Searches were limited to the English language due to constraints on available resources. The Population, Intervention, Control/Comparator, Outcome framework developed the initial search concepts. A review protocol was developed prior to searches, including the search strategies and terms in Supplementary Material 1, and the inclusion and exclusion criteria were detailed below.

### Study selection criteria

Inclusion criteria:

- Described or evaluated a method or process for eliciting and prioritizing ideas or topic areas for research and/or interventions for a defined community or geographic area
- Identified topic areas related to social determinants of health through a community focus beyond the individual level
- Primary research studies involving participants beyond researchers, such as members of the community or community organizations
- Undertaken in high-income countries based on World Bank classification

Exclusion criteria:

- Focussed solely on healthcare or organization and delivery of health services
- Focussed solely on individual behaviour change intervention
- Focussed solely on clinical research of specific health condition(s)
- Only included researchers without involving other groups or communities
- Studies undertaken in low- and middle-income countries
- Systematic reviews, scoping reviews, narrative reviews, and commentaries

### Study selection process

Screening was conducted using Rayyan software. Titles and abstracts of records retrieved from database searches were screened by one reviewer (J.B.) based on the study selection criteria to eliminate clearly irrelevant records. Full-text articles of the remaining studies were retrieved and independently assessed by two reviewers (J.B., C.S.) to make the final inclusion decisions. Discrepancies were discussed to reach consensus or by consulting a third reviewer (Y.-F.C.) for arbitration.

### Data extraction

A bespoke data extraction form (Supplementary Material 2) was developed, piloted, collaboratively discussed, and revised, then used by a team of 10 reviewers.

### Synthesis of results

An additional data charting form (Supplementary Material 3) was created for consistent classification to facilitate study comparisons. Data extraction and charting were undertaken by one reviewer and checked by another reviewer among the authors. Discrepancies were resolved through discussion. Given the descriptive nature of the scoping review, no risk of bias assessment was conducted.^18^ Extracted data were presented in tables to facilitate study comparisons. A narrative synthesis was adopted to present findings.

### Public involvement

Two public contributors, who were co-applicants involved in the HDRC’s implementation, were involved in the working group and provided public perspectives through the research process. Public contributors influenced, for example, additions to the data extraction form and collation of guiding principles, and extracted data from a sample of studies. Both contributors had been involved in research previously; neither had been involved in an evidence review. Further detail is reported using GRIPP 2 in Supplementary Material 4.^19^

## Results

### Screening

**Figure 1:**
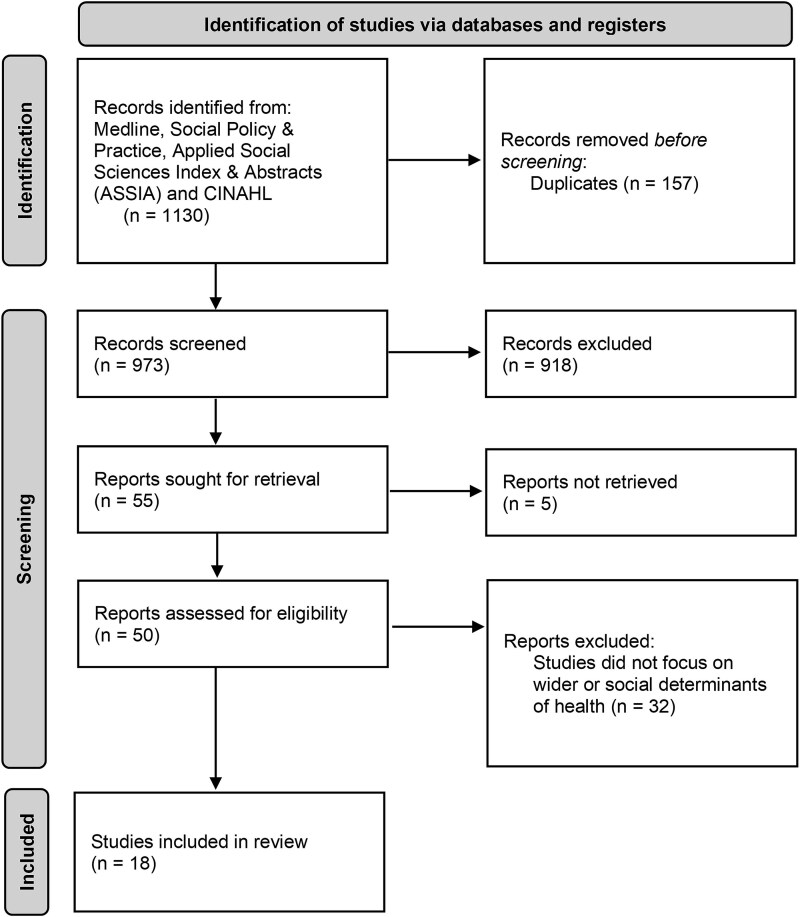
PRISMA flow diagram illustrating selection of studies at each stage of the review.

In June 2024, 973 records were reviewed for title and abstract screening and 55 were reviewed at full text. Five articles could not be retrieved, and other exclusions did not focus on the wider determinants of health or instead addressed, for example, healthcare. Following full-text eligibility screening, 18 studies were included (Fig. 1).

### Characteristics of included studies

#### Location and target population

The 18 included studies were conducted in seven different countries, mostly (*n* = 9) in the United States of America (USA) and states listed in Table 1.^20–28^ Three studies were conducted in the UK,^29–31^ two in Australia^32^^,^^33^ and one in each of New Zealand,^34^ Denmark,^35^ Japan,^36^ and Germany.^37^

**Table 1:** Key characteristics of included studies.

Study	Location and targeted population	Topic of interest	Aims and objectives	Conceptual framework, approach, or theoretical underpinning
Aadahl et al. 2023	Populations living in two municipalities in Denmark	Health and wellbeing interventions	To develop and implement prioritized health promotion interventions across sectors in municipalities and local communities that involve and empower citizens.	Super-setting approach with bi-directional bottom-up and top-down approaches
Ablah et al. 2016	Older, central core of Wichita, USA. Primarily low-to-middle income people.	Environmental concerns around toxic pollutants and risk reduction	To identify and prioritize the community’s environmental concerns through engaging community members in project design and establishing a community-based environmental leadership council.	Community Based Participatory Research (CBPR)
Addison et al. 2019	Members of the Equal North Network in the North of England, UK.	Reduction of health inequalities	To understand priorities for action in reducing health inequalities, and consensus building for how research can address these priorities, involving researchers, policy makers, and practitioners.	Delphi exercise
Akintobi et al. 2018	Low-income households, primarily African Americans, USA.	Evaluation of participatory methods to inform health research and interventions	To identify health needs and priorities to inform research and intervention implementation through qualitative and quantitative primary and secondary data.	CBPR
Bateman et al. 2017	Residents of Birmingham, Alabama, USA	Tackling social health determinants through collaborative partnership	To describe the process of identifying and prioritizing perceptions of neighbourhood characteristics (e.g. social cohesion, neighbourhood problems) by a community-academic coalition team and to inform local health initiatives.	CBPR
Brown et al. 2008	Black community served by South Florida Health Centre, USA	Health disparities and interventions	To explore the context of health disparities in the community, the composition of the community, what interventions may be effective in reducing health disparities, and the appropriate resources, informants, or data to answer these questions in the time available.	Participatory Action Research
Cartwright et al. 2023	Children and young people in the city of Bradford, UK	Research priorities for children and young people’s health and happiness	To co-produce research priorities for happy and healthy children and young people, and report the community-led research priority setting exercise	Modified James Lind Alliance process
Chung-do et al. 2019	Native Hawaiian rural community of Waimānalo	Community research priorities and community-academic research partnership	To identify community priorities for research and programme initiatives through strengthening relationships within the Waimānalo community and between community and academic researchers.	CBPR including a modified Nominal Group Technique
Doolan-Noble et al. 2018	Older adults in five cities in New Zealand	Research priorities for older adults	To conduct priority setting workshops to inform resource allocation to areas of highest research priority.	Co-production approach
Goold et al. 2018	Minorities and medically underserved communities in Michigan, USA	Evaluation of a health research prioritization process with minority and medically underserved communities	To evaluate the structure, process, and outcomes of a health research prioritization process using a serious game called CHoosing All Together (CHAT).	Deliberative exercise using a serious game
Haya et al. 2020	Rural community in Japan	Community health programmes	To collaborate and assess the community’s health needs and strengths and develop a tailored community health programme.	CBPR
Hoekstra et al. 2023	Public health researchers, practitioners, professionals, and public representatives in Germany	Public health research priorities	To identify public health priority research topics using a structured process, and to identify similarities and differences between groups.	Modified Delphi exercise
Iqbal et al. 2022	British Pakistani women living in deprived areas of Bradford, UK	Research priorities relating to obesity and Pakistani women, including influence of the social determinants of health	To determine the top 10 obesity health priorities for Pakistani women in Bradford and identify the related research priorities.	Feminist participatory action research (FPAR)
Israel et al. 2001	Residents living in the east and southwest area of Detroit city, USA	Health problems affecting residents and community-academic research partnership.	To describe and analyse the process of establishing, implementing, and evaluating a community-academic partnership focussed on health.	CBPR
Kreuter et al. 2012	Residents of five contiguous neighbourhoods in Atlanta, USA	Priority health and social or environmental problems and actions	To examine the effectiveness of a programme that aimed to elicit resident input to identify priority health and social or environmental problems, and prompt community actions to address those problems.	CBPR including photovoice
Massi et al. 2023	Aboriginal and Torres Strait Islanders	Health research priorities relating to preconception, pregnancy, postpartum, and early childhood.	To collaboratively identify health and medical research priorities for Indigenous young families relating to preconception, pregnancy, postpartum, and early childhood.	Participatory Action Research
Rideout et al. 2013	Population and communities in New York City, USA	Disparities in New York	To develop and implement a process for identifying community-defined priorities and areas for collaboration that could reduce disparities.	CBPR and Delphi exercise
Rikkers et al. 2015	Children living in West Australia	Research priorities for children’s health	To assess methods (cold calling and Community Conversations) for obtaining community views and priorities for child health research.	Comparison of ‘cold calling’ telephone survey approach with Community Conversations (public discussion forum)

Studies focussed on various target populations (Table 1) and involved communities of the location being studied. Many studies described the geographical area as having high deprivation and/or focussed on involving low-income groups.^20–31^ In addition, six studies specifically focussed on ethnic minority and/or indigenous groups,^21^^,^^23^^,^^24^^,^^28^^,^^31^^,^^32^ two studies focussed on children and young people,^30^^,^^33^ two studies focussed on women,^31^^,^^32^ and one focussed on older adults.^34^

### Key topics, aims, and approaches

#### Topics

Studies focussed on identifying, understanding, and/or prioritizing activities in relation to the social determinants of health, health inequalities, and/or community approaches (Table 1). Specific topic areas within the social determinants of health included environmental concerns^20^^,^^26^ and topics related to children and/or families.^30^^,^^32^^,^^33^

#### Aims

Studies aimed to identify priorities for research^21^^,^^23^^,^^24^^,^^26^^,^^29–34^^,^^37^ and/or health intervention priorities transformed into action.^20–23^^,^^25–29^^,^^31^^,^^35^^,^^36^ Four studies aimed to report findings from evaluating prioritization activities.^24–26^^,^^33^ Priority setting appeared to be part of wider work in three studies,^23^^,^^25^^,^^28^ and two studies^22^^,^^29^ intended to sustain priority setting partnerships for future community engagement.

### Study designs and approaches

A commonly reported approach to the prioritization process was Community-Based Participatory Research (CBPR)^20–23^^,^^25–27^^,^^36^ or Participatory Action Research.^28^^,^^31^^,^^32^ These approaches were described as community-driven, with active collaboration involving researchers, professionals or practitioners, and community members throughout. Other study designs included Delphi exercises to gather expert opinion through rounds of surveys.^27^^,^^29^^,^^37^ One study reported a super-setting approach that coordinated engagement of groups in multiple community settings,^35^ and another study used a nominal group technique of iterative discussions through gatherings^23^ A deliberative exercise using a serious game to promote informed, reasoned conversation about decisions was also reported,^24^ plus a modified James Lind Alliance approach^30^ that brought together children, professionals, and researchers to identify and prioritize research questions. Other articles did not specify a named approach but described principles of coproduction^34^ or evaluation.^33^ Further information about the steps of community engagement and prioritization is outlined in Table 2.

**Table 2:** Steps of community engagement and prioritization covered in each article**.**

Study	Identifying and engaging target populations	Identification and collection of ideas	Refining and collating ideas	Ranking ideas	Translating ideas into action plans	Implementing action plans	Evaluation of the priority setting process	Further activities & sustainability	Frequency^a^
Aadahl et al. 2023	Y	Y	Y	Y	Y	Y	N	Not reported	One-off
Ablah et al. 2016	Y	Y	Y	Y	N	N	N	Not reported	One-off
Addison et al. 2019	Y	Y	Y	Y	N	N	N	Y	One-off
Akintobi et al. 2018	Y	Y	Y	Y	Y	Y	N	Not reported	One-off
Bateman et al. 2017	Y	Y	Y	Y (method not explicitly described)	Y	Y	N	Y (coalition maintains efforts to build its infrastructure by pursuing grants and new resource opportunities)	One-off (although potential for coalition to repeat exercise)
Brown et al. 2008	Y	Y	Y	N	Y (Results informed recommendations for action)	Y (published elsewhere)	N	Y (Brief mention of subsequent projects and activities)	Ongoing
Cartwright et al. 2023	Y	Y	Y	Y (but did not conduct the final consensus workshop to agree ‘top 10’ due to COVID-19	N	N	N	N	One-off
Chung-do et al. 2019	Y	Y	Y	Y	Y	Y	Y	Y	Ongoing
Doolan-Noble et al. 2018	Y	Y	Y	Y	N	N	N	N	One-off
Goold et al. 2019	Y	Y	Y	Y	N	N	Y	N	One-off
Haya et al. 2020	Y	Y	Y	Y	Y	Y	N	N	One-off
Hoekstra et al. 2023	Y	Y	Y	Y	N	N	N	N	One-off
Iqbal et al. 2022	Y	Y	Y	Y	N	N	N	N	One-off
Israel et al. 2001	Y	Y	Y	Y	Y (but process not described)	Y (but process not described)	Y	Y	Ongoing
Kreuter et al. 2012	Y	Y	Y	Y	Y	Y	Y	N	One-off
Massi et al. 2023	Y	Y	Y	Y	N	N	N	N (reference as potential idea but no concrete plans)	One-off
Rideout et al. 2013	Y	Y	Y	Y	Y	N	N	N	One-off
Rikkers et al. 2015	Y	Y	N	N	Y (through survey)	N	Y	N	One-off

*Note*. Y = yes this was reported by the study. N = no, this was not reported by the study.

a At the time when the original article was written up.

### Methods and approaches to the prioritization process

#### Team coordinating the process

Eleven priority setting studies^20–26^^,^^28^^,^^30^^,^^35^^,^^36^ described multi-disciplinary teams or a steering committee of academic, nonprofit, community and/or health partners. Involving community members or leaders in guiding the project was considered important to engage communities with cultural responsiveness (Table 3). In studies adopting participatory methods, responsibilities for planning and coordination were often shared across individuals involved or not clearly delineated in the article. Some studies^25^^,^^27^^,^^35^^,^^36^ designated individuals with coordination and administration responsibilities so that community partners with limited capacity were not overburdened, such as employment of a local coordinator,^35^ or assignment of procedural tasks to university employees.^25^^,^^36^ Projects predominantly coordinated by research teams were also described^29^^,^^31–34^ with activities in managing and analysing data and coordinating community involvement.

**Table 3:** Aspects of the process related to organization, groups engaged, and methods and techniques for priority setting.

Study	Team coordinating the process and activities	Groups engaged and approaches to engagement	Methods and techniques for priority setting
Aadahl et al. 2023	The conceptual model allows the project team structure to be flexible (e.g. project researcher, community representatives), though with an employed local coordinator to provide administrative and technical support.	Across phases, groups involved were from (1) local municipality administration departments and high-level representatives, directors and elected council members, as well as (2) representatives from organizations, institutions, and associations from the public sector, private sector, and the civil society as community-based organizations. Eligibility and relevance of representatives from community-based organizations were determined by partners in the public administration.	In Phase 1, a local government-focussed workshop was delivered that aimed to jointly identify a thematic focus area and a target group for preventive intervention. In Phase 2, a community-focussed workshop was organized for representatives from community-based organizations relevant to the thematic focus area and target group to identify a variety of ideas and topics for action. In Phase 3, community action groups were formed with project staff and participants from the workshop across public private, and civic affiliations to develop and implement activities.
Ablah et al. 2016	University researchers and a design team of nine community leaders from nonprofit community groups, local government, industry, and academic institutions, plus 25 community members who assisted with organizing community concerns and engaging participants.	Fifty-two discussion groups were held with 1500 community members in community locations (e.g. neighbourhood association, public meetings). Following these discussions and through email and website promotion, 25 community members from businesses, neighbourhood associations, and community groups nominated themselves or others to join the Environmental Leadership Council (ELC) to support the prioritization process.	Environmental concerns were identified through discussion groups and nominal group techniques with community members, leading to a 92-page list. The Environmental Leadership Council then categorized and prioritized these concerns, combining responses and stratifying them into air, water, and solid waste, resulting in 19 issues. The 19 issues were presented to the public via an educational campaign, and 769 community members ranked them on a five-point scale on criteria of risks to the environment, health, economy, urgency for action, and perception of community interest in addressing the concern.
Addison et al. 2019	The research team analysed data from workshops and a Delphi survey. One facilitator and one scribe were present at each workshop.	Members of the Equal North Partnership, a research network of academics, policy, and practice members, were invited to join the study via events, email distribution lists, and social media. In total, 368 members were involved (46% practitioners, 54% academics; 73% female; 38% from the North East, 35% Yorkshire and Humber, 21% from the North West, and 6% not regionally based)	In round 1 of the Delphi exercise, two workshops (*n* = 190) and a survey (*n* = 63) were conducted to generate items related to inequalities. In round 2, an online survey invited participants (*n* = 144) to rank items, and in round 3, re-rating was conducted via an online survey after the median group result was known (*n* = 76). A third workshop was then conducted to triangulate results (*n* = 75)
Akintobi et al. 2018	Board of residents (*n* = 16), academic institutions (*n* = 3) and health/social service agencies (*n* = 4) who reviewed, monitored, and evaluated the activities, with a Data Monitoring and Evaluation Committee (*n* = 8; academic-community co-leadership) who prepared the results. Board members and research centre staff administered the surveys following training.	A total of 361 residents were recruited to the survey via convenience sampling, email, and social media. The Community Coalition Board members (involving neighbourhood residents, academic institutions and health/social service agencies) and other community leaders received training and recruited community members through their networks (e.g. via face-to-face neighbourhood meetings, recreational facilities, senior centres, and health clinics). Nonmonetary incentives were given to participants surveyed in person, with a small proportion conducted online.	A 30-question needs assessment survey with open and closed questions was updated and piloted for community members to identify perceived causes and solutions associated with community health profiles. Data from secondary sources (e.g. local health departments, community organizations, partner agencies) were also mined to inform development of a community profile (e.g. demographics and major causes of morbidity). Results were integrated and presented to the Community Coalition Board to coordinate subsequent action.
Bateman et al. 2017	Community coalition-academic team including a community engagement specialist and academic investigators working with neighbourhood association president and other community leaders. The coalition formed a community project committee and a development committee to implement programmes and sustain the coalition.	Project staff recruited community leaders via email, telephone, face-to-face meetings, and snowball methods to identify further community leaders who then formed a Community Coalition. The coalition informed a survey developed with academic investigators. Community members (*n* = 90) attending a coalition-hosted ‘get to know your neighbour’ event completed the survey. Nonmonetary incentives (t-shirts) were given to participants.	The Community Coalition met to discuss causes of health disparities and vision for a healthier neighbourhood, prioritizing the list of community concerns to address. These concerns informed the survey distributed to community members, which explored perceptions of stability, satisfaction, social/physical characteristics, and social cohesion in the neighbourhood, explored whether these aligned with the coalition’s concerns, and solicited ways to address those concerns. The survey results informed priority areas for health initiatives.
Brown et al. 2008	Multidisciplinary team including principal investigator and research team, health professionals, and a field team with resident cultural guides and community activists who assisted in gaining access to participants.	The project team recruited a field team of ethnographers, cultural guides, and a multidisciplinary health team, who then recruited participants (community members, staff in community-based organizations, health centres, and administrators) to data collection and a community advisory committee. A third and final committee meeting was held in a town hall to maximize community input.	A field team received training and then conducted interviews and focus groups to understand the contexts of health disparities. Secondary data sources were analysed to determine demographic characteristics associated with health outcomes. Presentations of the integrated interim findings were made to the community advisory committee, who provided interpretation and guidance throughout the iterative process. Following team consensus, a report was distributed to policymakers and key groups.
Cartwright et al. 2023	The project was initiated by the Born in Bradford (birth cohort research) team, who convened a multi-disciplinary, multi-ethnic community steering group to co-produce the project. Project team implementing the plan involved a principal investigator, research programme leads, public health specialist, study co-ordinator, research fellow, and undergraduate and postgraduate students.	A project team of academic and public health professionals and students was formed, plus a 12-member multidisciplinary steering group purposefully invited from a mix of professionals, faith, parents, voluntary and community sector, and lay representatives. The survey was open to all ages (but most suitable for adults) with no incentive, and promoted across social media, newsletters, flyers, local radio, and newspapers, and circulated through the steering groups’ networks. Paper surveys were available in clinics, public events, and shopping locations. For community members who did not speak English as a first language, face-to-face sessions were conducted, and schools were visited to engage young people. In total, 588 participants responded.	In the modified James Lind Alliance (JLA) method, a steering group was established that developed and piloted a survey. The survey was completed by 588 community members, which the steering group then used to agree a set of research questions. Shared priorities were then co-produced in a community-based workshop (*n* = 14) and meetings with the community steering group and community members. Finally, a total of 17 research questions were reviewed and amended by the steering group to make them easily understandable to the public.
Chung-do et al. 2019	Community-academic research partnership developed organically and over time, starting initially between two individuals.	Community members from local civic and community groups and any local resident interested in learning more about research were invited to meetings, with between 15 and 50 people over the initial six gatherings. Efforts (nonspecified) were made to encourage community members who were not affiliated with any institutions as well as young people and elders to promote multi-generational learning. Academic researchers and students involved in past programs and with existing relationships to the community were also invited.	Monthly gatherings were set up to bring together community members and academic researchers and students to share a meal and have an open discussion to identify community preferences and priorities for research and programmes, and to strengthen relationships between the community and researchers. Over time, iterative discussions involved using a modified nominal group technique to elicit many ideas, which were then shared, discussed, and voted on at the following meetings until consensus was reached on the top three priorities.
Doolan-Noble et al. 2018	Roadshow research team captured and considered information from the roadshows, including outputs by a graphic illustrator.	Groups including researchers, healthcare and service providers, and representatives from community-dwelling older adults, nongovernment organizations and Māori, and Pacific providers were invited to attend roadshow workshops. Attendees (*n* = 133) were recruited through personal invitations, flyers distributed via District Health Boards and social network adverts.	Workshops were delivered involving nominal group techniques to guide group discussions on key issues for older adults. Subgroup discussions were captured on paper and fed back to the larger group. All participants were then given three sticky dots to vote for their priorities, with the votes counted separately by two researchers.
Goold et al. 2018	Process led by a steering committee comprised majority community leaders and leaders of research institutions.	Five hundred nineteen members of underrepresented and medically underserved communities were recruited to 47 focus groups via local advertising (newspapers, adverts, radio), posting and distribution of flyers through community-based organizations, and through personal contacts.	A deliberative exercise in the form of a serious game (Choosing All Together [CHAT]) aimed to promote informed, reasoned dialogue and be credible and comprehensible to a lay audience. CHAT was an interactive game board on a tablet device, where participants allocated 50 markers to 92 potential options across four rounds. Participants set priorities as individuals (round 1), in groups of two to four (round 2), as a whole group (round 3), and again as individuals (round 4). After rounds 1 and 2, the group discussed scenarios about the consequences of their choices, and in round 3, deliberators articulated reasons for their priorities. In all rounds, trained facilitators asked deliberators to make fair decisions on behalf of fellow community members.
Haya et al. 2020	The research team collaborated with city officials and community partners, with a community advisory board discussing all plans and decisions. University researchers were responsible for all procedural tasks to reduce the burden for community partners, who bridged between the researchers and the community in recruiting participants.	The research team undertook initial community engagement to familiarize themselves with residents. A Community Advisory Board (*n* = 27) was set up, which included university researchers, a hospital doctor, community members, city officials, and community health office staff members. Community residents (*n* = 68) took part in a community forum, advertised through flyers distributed to households and posters on community bulletin boards. Subsequently, a questionnaire was distributed to all households in the community, with 773 household responses.	A community forum (world café) was held, and facilitators were provided a flexible question guide for the discussion. Participants were divided into 10 groups of six or seven, with a facilitator in each group. Discussions were recorded, transcribed, and thematically analysed. A quantitative survey was then conducted for participants to score the importance of the health issues and proposed actions identified from the qualitative discussions. The priorities resulting from the survey were analysed, including subgroup comparisons.
Hoekstra et al. 2023	The research team in collaboration with the German Public Health Administration and researchers. An advisory board was formed that reviewed the design and proposed analyses and that did not participate in the Delphi exercise.	Members of the German Public Health Association and individuals with public health expertise were invited to join two workshops, with between 40 and 50 attendees at each. In the second scoping, five public health researchers and practitioners were invited to form an advisory board. In a Delphi process, an online questionnaire was distributed and public health organizations (*n* = 140) were invited to nominate up to three participants. Participants (*n* = 94) included administrators, general public, associations of health providers, and statutory health providers, and healthcare professionals.	The process involved a scoping stage and Delphi stage. In the scoping stage, expert insights were gathered during two workshops, and these workshops established the study framework including an advisory board. In the Delphi stage, an online questionnaire was distributed to potential participants to gather proposals for priority research topics and assessment criteria, and the results were coded and aggregated. A second online questionnaire then asked participants to rate the proposed research topics according to assessment criteria (four-point Likert scale), and the results were analysed per topic, criterion, and group.
Iqbal et al. 2022	The research team recruited and planned this study, which formed part of a wider bottom–up project to co-produce research agendas with British Pakistani women.	Multisectoral groups with an interest in obesity in the target population were recruited to a survey, via purposive and snowball sampling to ensure representation across different professional and community sectors. Participants were approached by email, and 159 responded. Pakistani women were invited to participate in a ranking exercise and recruited via purposive sampling and snowball sampling. Initial participants were invited via WhatsApp, and those who expressed interest were provided information via email, with 32 women completing the ranking forms.	The article reports a two-step process. First, a survey was conducted with multisectoral professionals who listed their three most significant research priorities in the areas of overweight and obesity in Pakistani women. This survey generated 31 statements. In the second step, Pakistani women participated in a ranking exercise. Due to COVID-19, the exercise was conducted individually rather than in a group and delivered by trained peer researchers. Participants were asked to prioritize the top 10 statements most important to reduce obesity rates locally for Pakistani women, and scores were then collated and statements ranked.
Israel et al. 2001	An existing community-academic research centre with a board of representatives from the university, health, and community organizations. A project manager supported daily operations and communicating with partner organizations. Resources (e.g. staff) were important to establish and maintain partnership infrastructure.	Community-based organizations that had previously worked with the Michigan School of Public Health or the Detroit Health Department were contacted to take part in the Detroit Community-Academic Urban Research Centre. Meetings and discussion groups were held with organizations, and a board was set up to progress the work.	Following development of the board’s operations and principles, the board engaged in facilitated discussions. Participants first considered a problem statement individually, then shared and collated their responses leading to a list of problem areas that were discussed in more depth. Involved organizations also presented about their organizations’ activities and priorities. Following a brainstorming session, 25 problem areas were identified, reduced to 8, and then reduced to three main priorities through board discussions and applying specified criteria.
Kreuter et al. 2012	Five-member advisory board involved leaders of two community-based organizations, a nonprofit foundation, a health department, and a public health professor was recruited to oversee the research programme. Community health workers were recruited and trained to link and listen to communities.	Six Community Health Workers were recruited and trained in the relevant methods. Representatives from 12 local not-for-profit organizations were also recruited and surveyed to understand social capital between the organizations. The Community Health Workers recruited over 200 residents to attend an initial health fair to raise awareness of local organizations and five community listening sessions took place, attended by between 20 and 30 residents at each. Following community engagement and data collection, over 100 community residents were invited to a meeting to review data and select priorities.	The article reports a prioritization exercise and retrospective evaluation of the exercise after 3 years. The prioritization methods included community listening sessions, where attendees were presented with local health data and provided anonymous feedback for discussion. Photovoice was also used to elicit resident views and feelings, with 20 residents trained, and medical records were analysed. These data sources were reviewed by 100 residents who participated in a dinner meeting, and a nominal group technique was used to identify priorities for action. The retrospective evaluation included interviews with administrative individuals and staff and a focus group with community health workers. The evaluation confirmed the CBPR approach resulted in tangible results and supported sustained action.
Massi 2023	Research team led by three women (one Aboriginal Research Officer and two non-Indigenous Researchers), experienced in qualitative research, and with a broader research team including Indigenous and non-Indigenous researchers. Community organizations involved to determine suitability of proposed methods, and study champions at each partner site to assist planning of research team visits.	The research team built relationships with community-controlled health services, initially via the Queensland Aboriginal and Islander Health Council, with a study champion at each site. Services were encouraged to invite community members and promote yarning groups through flyers.	The article reports a protocol for a qualitative design using yarning methodology. Yarning methods involve semi-structured interviews carried out in informal and relaxed discussions where the researcher and participant visit places of relevance and a relationship is built. In phase one (already conducted), yarning sessions were conducted involving 61 people to discuss health-related topics. In phase 2, data are thematically analysed to develop research priority themes. In phase three, a Delphi workshop returns the findings to communities, and a workshop is conducted to reach consensus on priorities.
Rideout 2013	Partnership staff provided logistical support to workgroups and researchers collected and analysed data, recognizing that activity participants did not have the capacity for this. An outside consultant was hired to facilitate retreat activities.	A Community Engagement and Population Health Research (CEPHR) was developed, a partnership of community members, research, health and service providers, community-based organizations, and policymakers. The CEPHR invited trusted community leaders to serve on a Community Advisory Board (CAB, *n* = 22), which guided the CEPHR. A Steering Committee (*n* = 21) of university and health departments also guided the CEPHR.	Two rounds of online Delphi questionnaires were conducted over 4 weeks to identify priority areas important to community advisory board members. Answers in round one were grouped and led to 21 priority areas presented in round two, where participants chose 10 areas and indicated their priority on a five-point scale. Alongside the Delphi exercise, a planning retreat was delivered with the steering committee to generate and refine a vision statement. The Delphi exercise and retreat outputs were then presented in a joint meeting. Here, a snow card technique was used to organize different ideas and identify areas where the collaboration could focus their resource and capacity on high-need areas.
Rikkers 2015	The university research team planned, collected, and analysed data.	Random digit dialling of landlines was conducted to carry out telephone surveys (*n* = 816). Community conversations were also conducted, with one group recruited through telephone interviews (*n* = 3), and another group recruited through a participation network (*n* = 11).	The study administered voluntary 15-minute telephone surveys to people using random digit dialling of landlines, and to people who first attended a community conversation. Respondents were asked to give their opinion on issues on a 10-point Likert scale, and the survey elicited reviews on future research priorities. The community conversations also involved presentations of education-related research projects and small group discussions, which included identification of priorities for future research.

#### Groups engaged and approaches to engagement

Groups engaged in the prioritization process included community members in 11 studies^20–26^^,^^28^^,^^33^^,^^35^^,^^37^ and a further 6 studies engaged both community and professional groups^27^^,^^30–32^^,^^34^^,^^36^ (Table 3). One study engaged professionals only, who were described as practitioners living in the area under study.^29^ Several studies reported recruiting to or developing a board or steering group to design, guide, and implement the research project from the beginning.^21^^,^^22^^,^^26–28^^,^^30^^,^^31^^,^^36^^,^^37^ Another study began by broadly engaging the community and then developed a community-based leadership council with subgroups,^20^ or developed a board to progress the work.^25^

Methods to recruit target groups (Table 3) to the prioritization process included meetings between researchers and community organizations,^22^^,^^23^^,^^25^^,^^28^^,^^36^ targeted emails,^21^^,^^22^^,^^29–31^^,^^33^^,^^37^ local advertising (e.g. newspaper, posters, radio adverts),^24^^,^^30^^,^^36^ distribution of flyers,^24^^,^^32^^,^^34^^,^^36^ distribution of household surveys,^36^ social media adverts,^21^^,^^29^^,^^30^^,^^34^ opportunistic face-to-face surveys,^21^ via personal or professional contacts,^24^^,^^29^^,^^31^^,^^34^^,^^37^ and random digit dialling of landlines.^33^

#### Methods and techniques for identifying and setting priorities

Various methods and techniques were used to identify priorities, and a combination of techniques was often involved (Table 3). The most common method, included in 12 studies, involved workshops to engage communities in generating a broad range of priorities.^20^^,^^23^^,^^26^^,^^27^^,^^29^^,^^30^^,^^32–37^ Five studies used surveys^27^^,^^29^^,^^33^^,^^36^^,^^37^ and three used further workshops^29^^,^^35^^,^^37^ to conduct further prioritization towards a reduced list. In other studies, prioritization techniques included surveys,^21^^,^^22^^,^^31^ voting,^23^ discussions among collaborators,^25^^,^^30^ and interviewing.^26^^,^^28^

#### Methods and techniques for actions following prioritization

Following the prioritization process, 10 articles had translated ideas or outcomes from the prioritization exercise into action plans,^21–23^^,^^25–28^^,^^33^^,^^35^^,^^36^ with 8 implementing these action plans^21–23^^,^^25^^,^^26^^,^^28^^,^^35^^,^^36^ (Table 2). Most actions were identified through focus groups, surveys, or community activities and led by the community, in addition to sharing with policymakers for adoption in strategy or policy.

### Guiding principles and lessons for successful prioritization

Studies suggested guiding principles to enhance the effectiveness and success of the prioritization process (shown per study in Supplementary Material 5). Frequently reported was the importance of embedding multiple, diverse, and purposive methods of recruitment and involvement to enhance inclusion and representation in the priority setting process. For example, including information in different written, video, or verbal formats and languages,^20^^,^^24^^,^^27^ where English-only provisions may limit accessibility.^30^ Inclusion of in-person and remote approaches also aimed to include those digitally excluded.^30^

Broad involvement of groups and communities was widely expected to be beneficial to diversify ideas, and one study specifically aimed to involve participants beyond existing groups organized around the topic area being explored.^20^ Grassroots approaches helped prioritize community needs, ideas, and concerns, rather than those of professionals or researchers.^20^^,^^23^^,^^27^^,^^31^^,^^32^{Citation} Activities that were convenient to prospective participants, such as meetings held in accessible community locations,^22^^,^^28^ also facilitated engagement. Despite efforts, however, some studies reported low response rates or self-selecting participants that may not have represented wider, diverse communities,^29^^,^^30^^,^^33^^,^^34^^,^^36^ and additional purposeful strategies and engagement with community leaders may be necessary.

Critical to a successful prioritization process was promoting equitable involvement and valuing different contributors, aligned with coproduction principles. Activities to build trust included ice breakers,^22^ sharing meals,^22^^,^^23^ researchers attending community activities,^36^ broad partner involvement^26^ and by recognizing the time that may be needed, particularly in cases of initial distrust.^25^ Some studies, however, shared challenges in achieving equity between partners from different backgrounds, including where conversations could lack balance across collaborators.^20^^,^^28^^,^^36^ Practical barriers were reported, such as challenges with sharing data across partners.^21^ Prioritization processes were supported when a co-learning environment was facilitated between partners,^20^^,^^23^ for example, training to facilitate this co-learning.^28^ Finally, it was considered important to recognize the prioritization process as a long-term investment that can and should evolve.^25^^,^^30^^,^^35^

## Discussion

### Main findings of this study

This scoping review identified 18 articles that report priority setting exercises with communities in relation to the social determinants of health. Whilst priority setting methods were heterogeneous, many studies described similar guiding principles and the importance of participatory and equitable processes, activities to build trust, and considerations for inclusive and accessible involvement of diverse communities. The studies provide learning that can be taken forward by organizations or collaborations aiming to involve communities in prioritization. Methods used to prioritize research and/or interventions varied across studies, though commonly included workshops to identify topics or concerns, refined and prioritized through ranking exercises, as well as surveys, Delphi exercises, discussions, and a serious game, consistent with wider priority setting literature.^38^

Many of the prioritization processes in this review adopted participatory approaches and/or principles of coproduction. The resulting benefits included building trust with communities, training members of the community in research, and increasing relevance of priorities. Studies that embedded co-production often explicitly intended to shift power from researchers to communities, contrasting consultation-type approaches where power remains with researchers.^6^

### What is already known on this topic

In wider discourse, successful research prioritization processes should be fair, legitimate, informed by evidence, transparent, and involve diverse groups.^39^ The inclusion of bottom-up approaches also supports and democratizes the prioritization of concerns or ideas by communities, rather than only those of professionals or researchers.^40^ Prioritization approaches can ultimately be tokenistic if priorities are then decided, for example, by funding opportunities or priorities of researchers.^41^ Failing to address power dynamics in research prioritization can also lead to tokenistic and unethical processes that lack meaning and relevance to communities.^31^ Developing knowledge of evidence-informed prioritization approaches is of significance to research collaborations, including HDRCs. HDRC Medway have reported using the Analytic Hierarchy Process to score proposed research questions, with involvement from local residents in shaping and conducting the process, and with plans to co-design processes for wider resident input.^42^

### What this study adds

This review provides implications and examples of community prioritization in high-income settings relevant for place-based collaborations. The findings suggest the benefits of involving communities throughout the research cycle. Inclusive community involvement^43^ was enhanced via diverse and purposive recruitment strategies, including online and in-person approaches, and sharing information in accessible and diverse formats,^20^^,^^24^^,^^27^^,^^30^ and activities locally convenient to community members.^22^^,^^28^ Equitable involvement was facilitated via nonmonetary incentives, meals, visibility at community events, and taking time to build trust and long-term relationships.^20^^,^^23^^,^^24^^,^^27^^,^^30^^,^^43^ Many approaches were developed and/or delivered in culturally sensitive ways, working with community leaders to ensure local relevance and appropriateness, and building rapport and connections with diverse groups.^43^^,^^44^

The examples and approaches can be taken forward by research collaborations to develop meaningful community prioritization processes. Collaborations, including HDRCs, should consider implementation of advisory boards that equitably represent diverse communities, to effectively target resources and improve the relevance and legitimacy of research.^45^ Embedding administration and coordination support via an employed or dedicated role may also reduce burden on community organizations.^35^ Similarly, involving facilitators to coordinate coproduction activities ensures the practical necessities and enables collaborators to focus on productive discussions.^46^

The findings may also apply to the development of national calls for evidence, areas of research interest for government,^47^ and priorities of research funders, ensuring that communities’ voices influence effective prioritization and allocation of resources. Ensuring time and financial resources to facilitate meaningful community prioritization will be critical to facilitate impactful research and interventions. Translating priorities into action should be considered throughout, including planning how to communicate priorities to researchers, professionals, and communities.^48^ Demonstrated through this article, there is value in transparently reporting the process and principles used to identify research priorities,^48^^,^^49^ with a relevant reporting checklist available.^39^

### Strengths and limitations

To our knowledge, this is the first formalized scoping review of prioritization approaches relating to the social determinants of health. Findings will benefit organizations planning priority setting activities with communities. Our team included reviewers from different professional backgrounds and public contributors, and multiple reviewers screened and extracted data. This process helped maximize the rigour, relevance, and usefulness of plans and steps in the review. Preliminary findings also provided timely recommendations in the development of our HDRC. Given these aims to influence early HDRC implementation and development, potential limitations are that search strategies such as forward citation searching were not performed, and only published studies in English were included. Critical appraisal was not conducted, and the quality or risk of bias of included studies was not assessed. Finally, the included studies took place in different nations, and there are considerations for transferability when applying findings to specific localized geographies. Considerations include the fact that local collaborations should work with community organizations and leaders to understand local contexts and co-produce appropriate and meaningful methods for their local communities.

## Conclusion

This is the first scoping review to synthesize knowledge about approaches and principles to conducting priority setting activities with communities and relating to the social determinants of health. Eighteen studies were included and together highlight a range of approaches to recruit and engage communities and determine and refine priorities, though with common themes around the benefits of co-produced and participatory approaches. Local and national collaborations which are seeking to develop priorities for research or action that are informed by communities should use the guiding principles and approaches highlighted in this review to ensure diverse, representative, and equitable representation from communities. Of particular importance is a coproduced approach to shift power from researchers to communities. We identified several methods that can be used for community involvement in priority setting, ensuring that future research can select the most appropriate approach for the communities involved.

## Supplementary Material

Supplementary_file_fdaf151

## Data Availability

Data sharing is not applicable to this article as no new data were created or analysed in this study.

## Notes

**Catherine E. Shuttleworth**, Public Health Specialty Registrar

**Jack M. Birch**, Research Associate

**Lauren Bell**, Research Fellow

**Michael Ogunyemi**, Research Assistant

**Cameron D. Ley**, Research Fellow

**Harmony Lully**, Programme Officer

**John Wilcox**, Head of NIHR Coventry Health Determinants Research Collaboration (HDRC)

**Richard Grant**, Public Contributor

**Jane Whitehouse**, Public Contributor

**Naila Dracup**, Information Specialist

**Sophie Staniszewska**, Professor of Health Research (Patient and Public Involvement and Experiences of Care)

**Yen-Fu Chen**, Associate Professor in Evidence Synthesis and Health Services Research
