# Supplementary material for: Approaches for community intervention and research priority setting to reduce health inequalities: a scoping review
Source: J Public Health (Oxf). 2025 Dec 4;48(1):70–86. doi: 10.1093/pubmed/fdaf151 (PMC13017089; doi:10.1093/pubmed/fdaf151)
Supplement: Supplementary_file_fdaf151 [file supplementary_file_fdaf151.docx]

**Supplementary File**

**Supplementary Material 1**

Full search strategies

**Ovid MEDLINE(R) ALL 1946 to May 22, 2024**

| Concept | # | Search terms | Hits |
| --- | --- | --- | --- |
| Community involvement or health/ wider determinants | 1 | Population Groups/ and (Involve* or participat* or engagement* or empower* or powered).ti,ab,kf. | 733 |
|  | 2 | (((Under-represented or underrepresented or underserved or ethnic* or deprivation or deprived) adj2 (group* or stakeholder* or consumer* or area* or participant* or communit* or public)) and (involve* or participat* or engagement* or empower* or powered)).ti,ab,kf,kw. | 10119 |
|  | 3 | (((Seldom heard or hard-to-reach) adj3 (group* or communit* or population*)) and (involve* or participat* or engagement* or empower* or powered)).ti,ab,kf,kw. | 569 |
|  | 4 | (((Population or national* or local* or communit* or public) adj1 level*) and (involve* or participat* or engagement* or empower* or powered)).ti,ab,kf,kw. | 11538 |
|  | 5 | Patient Participation/ | 29960 |
|  | 6 | Community Participation/ | 18654 |
|  | 7 | Stakeholder participation/ | 2220 |
|  | 8 | (Co-produc* or coproduc* or co-design* or co-creat*).ti,ab,kf. | 14074 |
|  | 9 | ((Patient and public involvement) or PPI or PPIE).ti,ab,kf. | 32279 |
|  | 10 | (Person-centred* or person-centered*).ti,ab,kf. | 11493 |
|  | 11 | ((Health or wider) adj2 determinant*).ti,ab,kf. | 22062 |
|  | 12 | or/1-11 [Community involvement or health/ wider determinants] | 148469 |
| Priority setting | 13 | Community-Based Participatory Research/ | 5841 |
|  | 14 | Health Priorities/ | 11548 |
|  | 15 | ((Health or wellbeing or well-being or social* or care or setting or communit*) adj2 (priority or priorities or prioriti?ation)).ti,ab,kf,kw. | 16389 |
|  | 16 | (Regional health planning/ or community health planning/) and priorit*.ti,ab,kf. | 478 |
|  | 17 | or/14-16 [priority setting] | 25769 |
| Research | 18 | Research/ | 205152 |
|  | 19 | Health Services Research/ | 38712 |
|  | 20 | Research Design/ | 128109 |
|  | 21 | Research.ti,kf. | 421749 |
|  | 22 | Research.ab. /freq=2 | 455575 |
|  | 23 | (Evidence adj2 (base* or gap*)).ti,ab,kf,kw. | 201003 |
|  | 24 | or/18-23 [Research] | 1195579 |
| Methods | 25 | methods/ | 231755 |
|  | 26 | "Systematic Review"/ | 261192 |
|  | 27 | Systematic Reviews as Topic/ | 13216 |
|  | 28 | review*.ti. | 775580 |
|  | 29 | review.pt. | 3325301 |
|  | 30 | (guideline or practice guideline or consensus development conference or consensus development conference, NIH).pt. | 48411 |
|  | 31 | guidelines as topic/ or practice guidelines as topic/ | 170148 |
|  | 32 | Practice Guideline/ | 31433 |
|  | 33 | Checklist/ | 9149 |
|  | 34 | Consensus/ | 22834 |
|  | 35 | Delphi Technique/ | 9385 |
|  | 36 | Decision Making/ | 106200 |
|  | 37 | (guideline or guidance or guide or guiding or Tutorial or Tutorials or white paper or Framework or Checklist or Checklists or step-by-step or Primer or pitfall or Pitfalls or consensus* or Delphi or Expert-panel or toolkit*).ti,ab,kf. | 1313218 |
|  | 38 | ((provide or providing or provided or provision or give or giving or gave or given or practical) adj2 (advice or recommend* or tip or tips)).ti,ab,kf,kw. | 42651 |
|  | 39 | Recommend*.ab. /freq=2 | 163865 |
|  | 40 | ((best or code or good) adj2 (practice or practices)).ti,ab,kf,kw. | 63244 |
|  | 41 | (guidelines or Standard or Standards or Recommend* or Elaboration or elaborating or Explanation or explaining or extension).ti,kf. | 284001 |
|  | 42 | (Statement* or Principle or Principles or Principled or tool* or Rule or Rules or how-to or critical-question* or approach* or exercise* or approach* or framework* or program* or checklist* or strateg* or process* or method* or project* or focus*).ti,kf. | 2556419 |
|  | 43 | or/25-42 [Methods] | 7334919 |
| Local community/ wider determinants and research and priorities and methods | 44 | 13 and 17 and 43 | 105 |
|  | 45 | 12 and 17 and 24 and 43 | 522 |
|  | 46 | 44 or 45 | 584 |
| Limiting to English language | 47 | limit 46 to english language | 576 |

**Social Policy & Practice**

1 ((Under-represented or underrepresented or underserved or ethnic* or deprivation or deprived) adj2 (group* or stakeholder* or consumer* or area* or participant* or communit*) adj3 (involve* or participat* or engagement* or empower* or powered)).ti,ab. 146

2 ((Seldom heard or hard-to-reach) adj3 (group* or communit* or population*) adj5 (involve* or participat* or engagement* or empower* or powered)).ti,ab. 37

3 (((Population or national* or local* or communit* or public) adj1 level*) and (involve* or participat* or engagement* or empower* or powered)).ti,ab. 1048

4 (Co-produc* or coproduc* or co-design* or co-creat*).ti,ab. 1919

5 ((Patient and public involvement) or PPI or PPIE).ti,ab. 227

6 (Person-centred* or person-centered*).ti,ab. 3190

7 ((Health or wider) adj2 determinant*).ti,ab. 719

8 or/1-7 7061

9 ((Health or wellbeing or well-being or social* or care or setting or communit*) adj2 (priority or priorities or prioriti?ation)).ti,ab. 871

10 Research.ti. 15310

11 Research.ab. /freq=2 21334

12 (Evidence adj2 (base* or gap*)).ti,ab. 8067

13 or/10-12 37800

14 8 and 9 and 13 15

**Applied Social Sciences Index & Abstracts (ASSIA)**

| Set# | Searched for | Databases | Results |
| --- | --- | --- | --- |
| S1 | TI,AB,KW(("Under-represented" or underrepresented or underserved or ethnic* or deprivation or deprived) NEAR/2 (group* or stakeholder* or consumer* or area* or participant* or communit*) NEAR/3 (involve* or participat* or engagement* or empower* or powered)) | Applied Social Sciences Index & Abstracts (ASSIA) | 167 |
| S2 | TI,AB,KW(("Seldom heard" or "hard-to-reach") NEAR/3 (group* or communit* or population*) AND (involve* or participat* or engagement* or empower* or powered)) | Applied Social Sciences Index & Abstracts (ASSIA) | 155 |
| S3 | TI,AB,KW(((Population OR national* OR local* OR communit* or public) NEAR/1 level*) near/3 (involve* OR participat* OR engagement* OR empower* OR powered)) | Applied Social Sciences Index & Abstracts (ASSIA) | 233 |
| S4 | MAINSUBJECT.EXACT("Patient participation") | Applied Social Sciences Index & Abstracts (ASSIA) | 1864 |
| S5 | TI,AB(Co-produc* or coproduc* or co-design* or co-creat*) | Applied Social Sciences Index & Abstracts (ASSIA) | 2164 |
| S6 | TI,AB,KW("Person-centred*" or "person-centered*" or ("person centred") or ("person centred")) | Applied Social Sciences Index & Abstracts (ASSIA) | 3835 |
| S7 | MAINSUBJECT.EXACT("Determinants") | Applied Social Sciences Index & Abstracts (ASSIA) | 686 |
| S8 | TI,AB,KW((Health or wider) near/2 determinant*) | Applied Social Sciences Index & Abstracts (ASSIA) | 3414 |
| S9 | [S1] OR [S2] OR [S3] OR [S4] OR [S5] OR [S6] OR [S7] OR [S8] | Applied Social Sciences Index & Abstracts (ASSIA)  These databases are searched for part of your query. | 12190 |
| S10 | MAINSUBJECT.EXACT("Priorities") | Applied Social Sciences Index & Abstracts (ASSIA) | 1269 |
| S11 | TI,AB,KW((Health or wellbeing or well-being or social or care) NEAR/2 (plan* or priorit*)) | Applied Social Sciences Index & Abstracts (ASSIA) | 12823 |
| S12 | ti,ab(("Priority setting" or prioritisation or prioritization) near/3 (exercise* or approach* or framework* or tool* or program* or approach* or checklist* or strateg* or process* or method* or project* or focus*)) | Applied Social Sciences Index & Abstracts (ASSIA) | 503 |
| S13 | ti("Priority setting" or prioritisation or prioritization) | Applied Social Sciences Index & Abstracts (ASSIA) | 485 |
| S14 | [S10] OR [S11] OR [S12] OR [S13] | Applied Social Sciences Index & Abstracts (ASSIA)  These databases are searched for part of your query. | 14242 |
| S15 | MAINSUBJECT.EXACT("Research") OR MAINSUBJECT.EXACT("Community research") | Applied Social Sciences Index & Abstracts (ASSIA) | 28707 |
| S16 | MAINSUBJECT.EXACT("Participatory research") | Applied Social Sciences Index & Abstracts (ASSIA) | 1659 |
| S17 | ti(Research) | Applied Social Sciences Index & Abstracts (ASSIA) | 38313 |
| S18 | ti,ab,kw(Evidence near/2 (base or based or gap or gaps)) | Applied Social Sciences Index & Abstracts (ASSIA) | 27750 |
| S19 | [S15] OR [S16] OR [S17] OR [S18] | Applied Social Sciences Index & Abstracts (ASSIA)  These databases are searched for part of your query. | 84751 |
| S20 | [S9] AND [S14] AND [S19] | Applied Social Sciences Index & Abstracts (ASSIA)  These databases are searched for part of your query. | 103 |
| S21 | [S9] AND [S14] AND [S19] | Applied Social Sciences Index & Abstracts (ASSIA)  These databases are searched for part of your query. | 103 |

**CINAHAL**

| **#** | **Query** | **Results** |
| --- | --- | --- |
| S1 | (MH "Population") OR (MH "Social Group") OR (MH "Urban Population") OR (MH "Suburban Population") OR (MH "Rural Population") | 29,537 |
| S2 | TI ( (((Under-represented or underrepresented or underserved or ethnic* or deprivation or deprived) N2 (group* or stakeholder* or consumer* or area* or participant* or communit* or public)) and (involve* or participat* or engagement* or empower* or powered)) ) OR AB ( (((Under-represented or underrepresented or underserved or ethnic* or deprivation or deprived) N2 (group* or stakeholder* or consumer* or area* or participant* or communit* or public)) and (involve* or participat* or engagement* or empower* or powered)) ) OR SU ( (((Under-represented or underrepresented or underserved or ethnic* or deprivation or deprived) N2 (group* or stakeholder* or consumer* or area* or participant* or communit* or public)) and (involve* or participat* or engagement* or empower* or powered)) ) | 5,092 |
| S3 | TI ( (((Seldom heard or hard-to-reach) N3 (group* or communit* or population*)) and (involve* or participat* or engagement* or empower* or powered)). ) OR AB ( (((Seldom heard or hard-to-reach) N3 (group* or communit* or population*)) and (involve* or participat* or engagement* or empower* or powered)). ) OR SU ( (((Seldom heard or hard-to-reach) N3 (group* or communit* or population*)) and (involve* or participat* or engagement* or empower* or powered)). ) | 306 |
| S4 | TI ( (((Population or national* or local* or communit* or public) N1 level*) and (involve* or participat* or engagement* or empower* or powered)). ) OR AB ( (((Population or national* or local* or communit* or public) N1 level*) and (involve* or participat* or engagement* or empower* or powered)). ) OR SU ( (((Population or national* or local* or communit* or public) N1 level*) and (involve* or participat* or engagement* or empower* or powered)). ) | 5,340 |
| S5 | (MH "Patient Participation") OR (MH "Stakeholder Participation") OR (MH "Consumer Participation") | 28,566 |
| S6 | TI ( (Co-produc* or coproduc* or co-design* or co-creat*) ) OR AB ( (Co-produc* or coproduc* or co-design* or co-creat*). ) OR SU ( (Co-produc* or coproduc* or co-design* or co-creat*). ) | 5,174 |
| S7 | (MH "Social Determinants of Health") | 12,889 |
| S8 | TI ( ((Health or wider) n2 determinant*) ) OR AB ( ((Health or wider) n2 determinant*) ) OR SU ( ((Health or wider) n2 determinant*) ) | 20,377 |
| S9 | S1 OR S2 OR S3 OR S4 OR S5 OR S6 OR S7 OR S8 | 91,245 |
| S10 | (MH "Health Priorities") | 793 |
| S11 | TI ( ((Health or wellbeing or well-being or social* or care or setting or communit*) n2 (priority or priorities or prioriti?ation)). ) OR AB ( ((Health or wellbeing or well-being or social* or care or setting or communit*) n2 (priority or priorities or prioriti?ation)). ) OR SU ( ((Health or wellbeing or well-being or social* or care or setting or communit*) n2 (priority or priorities or prioriti?ation)). ) | 10,537 |
| S12 | TI priorit* OR AB priorit* | 67,072 |
| S13 | (MH "Health and Welfare Planning") | 11,881 |
| S14 | S12 AND S13 | 1,629 |
| S15 | TI ( (("Priority setting" or prioritisation or prioritization) N3 (exercise* or approach* or framework* or tool* or program* or approach* or checklist* or strateg* or process* or method* or project* or focus*)) ) AND AB ( (("Priority setting" or prioritisation or prioritization) N3 (exercise* or approach* or framework* or tool* or program* or approach* or checklist* or strateg* or process* or method* or project* or focus*)) ) OR SU ( (("Priority setting" or prioritisation or prioritization) N3 (exercise* or approach* or framework* or tool* or program* or approach* or checklist* or strateg* or process* or method* or project* or focus*)) ) | 121 |
| S16 | TI ((("Priority setting" or prioritisation or prioritization))) | 1,526 |
| S17 | S10 OR S11 OR S14 OR S15 OR S16 | 12,131 |
| S18 | (MH "Research") | 31,787 |
| S19 | (MH "Health Services Research") | 16,843 |
| S20 | (MH "Study Design") | 37,405 |
| S21 | TI research OR AB Research | 714,091 |
| S22 | TI ( (Evidence n2 (base* or gap*)) ) OR AB ( (Evidence n2 (base* or gap*)) ) OR SU ( (Evidence n2 (base* or gap*)) ) | 159,318 |
| S23 | S18 OR S19 OR S20 OR S21 OR S22 | 876,874 |
| S24 | (MH "Research Priorities") | 4,269 |
| S25 | S9 AND S17 AND S23 | 547 |
| S26 | S9 AND S24 | 260 |
| S27 | S25 OR S26 | 737 |
| S28 | (MH "Systematic Review") | 0 |
| S29 | (MH "Systematic Review") | 134,286 |
| S30 | TI review* | 315,676 |
| S31 | (MH "Practice Guidelines") OR (MH "Checklists") OR (MH "Consensus") OR (MH "Delphi Technique") or (MH "Decision Making") | 197,679 |
| S32 | TI ( (guideline or guidance or guide or guiding or Tutorial or Tutorials or white paper or Framework or Checklist or Checklists or step-by-step or Primer or pitfall or Pitfalls or consensus* or Delphi or Expert-panel or toolkit*) ) OR AB ( (guideline or guidance or guide or guiding or Tutorial or Tutorials or white paper or Framework or Checklist or Checklists or step-by-step or Primer or pitfall or Pitfalls or consensus* or Delphi or Expert-panel or toolkit*) ) OR SU ( (guideline or guidance or guide or guiding or Tutorial or Tutorials or white paper or Framework or Checklist or Checklists or step-by-step or Primer or pitfall or Pitfalls or consensus* or Delphi or Expert-panel or toolkit*) ) | 654,535 |
| S33 | TI ( ((provide or providing or provided or provision or give or giving or gave or given or practical) n2 (advice or recommend* or tip or tips)). ) OR AB ( ((provide or providing or provided or provision or give or giving or gave or given or practical) n2 (advice or recommend* or tip or tips)). ) OR SU ( ((provide or providing or provided or provision or give or giving or gave or given or practical) n2 (advice or recommend* or tip or tips)) | 22,835 |
| S34 | TI ( ((best or code or good) n2 (practice or practices)). ) OR AB ( ((best or code or good) n2 (practice or practices)). ) OR SU ( ((best or code or good) n2 (practice or practices)). ) | 34,173 |
| S35 | TI ( (guidelines or Standard or Standards or Recommend* or Elaboration or elaborating or Explanation or explaining or extension). ) OR SU ( (guidelines or Standard or Standards or Recommend* or Elaboration or elaborating or Explanation or explaining or extension). ) | 351,697 |
| S36 | TI ((Statement* or Principle or Principles or Principled or tool or tools or Rule or Rules or how-to or critical-question* or approach* or exercise*)) | 270,075 |
| S37 | S28 OR S29 OR S30 OR S31 OR S32 OR S33 OR S34 OR S35 OR S36 | 1,428,892 |
| S38 | S27 AND S37 | 402 |
| S39 | S27 AND S37 | 400 |

**Carrot2**

"research priorities" "local community" 90 result 36- screened for relevance includes

**Supplementary material 2**

**Data extraction form**

Whilst conducting the data extraction, here are some points to keep in mind:

- What questions are we going to ask – issues of concerns, solutions, or exact research questions?
- Methods for reaching out and collecting ideas
- The order of involvement of different stakeholders and nature (e.g. ongoing; periodical or ad hoc)
- Methods for collating and translating ideas into research areas/questions
- Timing, criteria and methods for prioritisation (ranking)
- Methods for evaluation of the process
- Guiding principles for the process

| **Data extraction item** | **Content** |
| --- | --- |
| 1. Paper details    1. Lead author surname    2. Year published |  |
| 1. Why was research prioritisation completed?    1. Population of interest    2. Overarching topic to be studied |  |
| 1. What was the research prioritisation process?    1. Was a named process used (e.g., James Lind Alliance)?    2. How was the research prioritisation process described in the paper?    3. What methods did they use? (E.g., Surveys, Delphi, interviews) |  |
| 1. When was the research prioritisation conducted?    1. Between which dates was the process completed?    2. How long was the process in total? |  |
| 1. Where did the study take place?    1. Country    2. City |  |
| 1. Resources required for research prioritisation process    1. Who was involved in the research prioritisation?       1. Professions, expertise etc    2. Was it conducted with in-person meetings or remote meetings? |  |
| 1. How were the public involved in the process? (Co-producing, co-designing, engaging, consulting, informing, educating, coercing)    1. To what extent was the involvement – e.g., consultation, co-production.    2. How were they recruited? |  |
| 1. Do the priorities identified in the process match the aims stated prior to the process? |  |
| 1. Other comments   (e.g., any additional learning on the process captured in the paper. Did the authors mention what they could have done differently? Any challenges? Successes?) |  |

**Supplementary Material 3 – Secondary Data Extraction Form**

| Study | Identifying and engaging target populations | Identification and collection of ideas | Refining and collating ideas | Ranking ideas | Translating ideas into action plans | Implementing action plans | Evaluation of the priority setting process | Further activities & sustainability | Frequency |
| --- | --- | --- | --- | --- | --- | --- | --- | --- | --- |
|  |  |  |  |  |  |  |  |  |  |

**Supplementary Material 4 – GRIPP 2 Short Form Checklist**

The GRIPP2 Short Form reporting checklist^19^ was used to report key items of the study where two public contributors from the Coventry HDRC supported the study.

1. **Aim**

The Coventry HDRC aims to involve the public so that it’s research can best impact on health determinants in Coventry. The HDRC involves two public contributors and they were involved in this scoping review with the aim of ensuring that the review provides research that is meaningful and relevant to local communities, as well as providing opportunities for the contributors to learn new skills in research.

1. **Methods**

The two public contributors were part of the working group that sought to understand how research priority setting might be conducted as part of Coventry HDRC. The group identified that it would be helpful to understand what methods for involving communities in research priority setting had been undertaken and therefore this scoping review was undertaken. Through a series of meetings and emails, the public contributors were involved in the design of the review (including search strategy and data extraction template), data extraction, reviewing findings and contributing to the discussion section of the paper. This helped to maximise the rigor, relevance, and usefulness of plans and steps in the review.

1. **Study results**

Both public contributors extracted data from 2 papers each as part of the review. For studies that met the eligibility criteria, this data was included in the write up of the results. This allowed the contributors to get further exposure of the research process and develop their skills in this area.

1. **Discussion and conclusions**

The public contributors had an equal level of power and influence as other members of the working group. This meant that their insights and suggestions were considered and, where appropriate, incorporated into the scoping review in the same manner as other members of the group.

1. **Reflections / critical perspective**

The review benefited from having public contributors to increase the diversity and depth of thought when planning and developing the review and will ultimately make it more meaningful to both the Coventry HDRC and other organisations that wish to involve communities in priority setting. It also allowed contributors to develop their skills in research, including opportunities to comment on versions of the paper. Future research projects and reviews could focus on working collaboratively to produce a paper that is written in an accessible way.

**Supplementary Material 5. Guiding principles and learning for successful prioritisation**

| **Study** | **Guiding principles and learning for successful prioritisation** |
| --- | --- |
| Aadahl 2023 | Nine factors for successful prioritisation were; 1) adoption is prioritised and considered a long-term investment; 2) formalised political approval and commitment by the public administration and local government; 3) local government politicians and high-level decision-makers from all sectors and departments are invited to jointly select the thematic focus area and primary target group for the intervention; 4) processes of selecting the thematic focus area and target group is informed by relevant available evidence and knowledge; 5) activities and projects complement existing local initiatives; 6) Contributors from the public sector, the private sector, and civil society are engaged equitably as co-owners in the process; 7) plans, processes, and developments are widely coordinated and communicated between departments in the public administration and between the public administration and community-based groups; 8) activities and projects include relevant strategies, plans, and measures to secure high levels of effectiveness, integration, and sustainability; 9) lessons learned are documented and used to define and support future interventions. |
| Ablah 2016 | Bottom-up grassroots approach helped to address community concerns without bias or institutional hindrance, and generalised community groups were approached rather than those organised around environmental concerns. Information and outputs aimed to be easily understandable and in different formats (e.g., written, video). Individuals from different backgrounds (e.g., community members, researchers) learned from each other in a facilitated co-learning environment, though there were challenges balancing academic conversations with a community desire for action. |
| Addison 2019 | The exercise was undertaken at a time of expanding the network, and so multiple methods of engagement and re-engagement were used. Participants were self-selecting with particular interest in health inequality and a heterogenous group of practice or academic experts, however a low survey response rate was received. |
| Akintobi 2018 | The process empowered community members to take on roles as researchers, which developed locally relevant research questions and expanded relationships with the community. A convenience sampling approach intended to recruit individuals not otherwise engaged in research, though may have limited generalisability.  The process also shaped the development of policy, systems, and approaches to community-driven data collection and health improvement, however there were challenges related to data sharing across partners. |
| Bateman 2017 | Ice breakers were implemented to build trust, and dinner was provided for coalition members. Collecting data from different groups and in different methods strengthened the interpretation of findings. Meetings were held in the community to minimise travel burden and monthly on a day and time most convenient for members, and communication between meetings was via email and Facebook postings. |
| Brown 2008 | Contributors were provided three days of training to support method development and delivery. Inclusion of cultural guides resulted in the best representation of community members, though some conversations were reported to be dominated by health service personnel. Final session was held in community location to maximise engagement. |
| Cartwright 2023 | A mix of remote and in-person approaches were to enhance inclusion, including for those digitally excluded. However, the survey was only available in English which may have limited accessibility, and children and young people were underrepresented in the surveys whereas older people were overrepresented. Despite attempts to reduce researcher influence, researchers may have influenced formulation of the survey questions. It was concluded that the activity should not be viewed in isolation, recognising research agendas are ongoing and dynamic. |
| Chung-do 2019 | The partnership brought people together to share a meal and “talk story”, with the direction of the partnership being open to allow for participatory and organic processes. Co-learning was encouraged, and the collaboration reported increasing their knowledge of research or Native Hawaiian values and practices. Community members were continuously encouraged to share feedback and reflections, so their voices were heard and prioritised, with an ethical infrastructure that promoted self-determination, community control, and trust. Protocols and Rules of Engagement were developed to support transparency and structure among the membership, where attendance and engagement can be fluid) |
| Doolan-Noble 2018 | Broad engagement of a heterogenous group intended to share ideas, although despite expansive recruitment, few Māori and Pasifika people attended the roadshows. Study informed by nine elements of good practice described by ^8^ throughout preparatory work (context, use of a comprehensive approach, inclusiveness, information gathering, planning for implementation), deciding on priorities (relevant criteria and methods on deciding principles) and after the priorities had been set (evaluation and transparency). |
| Goold 2018 | Purposeful recruitment approaches successfully recruited an over-representation of minority and low-income residents. Content during the process was designed for a lay audience, in English and Spanish, and began with a brief video to promote engagement. |
| Haya 2020 | Researchers participated in community activities to build trust. High presence of community partners on the advisory board strengthened representation, however study participants were most often older adults, with underrepresentation of younger people. Tasks were assigned to align to different contributors’ skills (procedural tasks for researchers, and community partners were responsible for engaging with communities). The participatory approach intended to achieve community ownership of the programme, increasing the likelihood of sustainability. |
| Hoekstra 2023 | Researchers balanced eliciting proposals from a wide range of groups, whilst ensuring the proposals were within scope. Purposive recruitment reduced costs and improved participation rates, with the modified Delphi technique intending to increase participation and inclusion of groups that may have otherwise been underrepresented. However, the Delphi process was conducted in less depth and may indicate preferences rather than a consensus. |
| Iqbal 2022 | Coproduction involved Pakistani women who translated the concerns from participants into research priorities to prevent misinterpretation. Purposive sampling was adopted to identify priorities and recruit non-English speaking women to support wider applicability and address power imbalance. |
| Israel 2001 | Equitable involvement in participatory research was guided by principles to (i) recognise the community as a unit of identity; (ii) build on strengths and resources within the community; (iii) facilitate collaborative partnerships in all phases of the research (iv) integrate knowledge and action for the mutual benefit of all partners (v) promote a co-learning and empowering process that attends to social inequalities (vi) involve a cyclical and iterative process (vii) address health from a both positive and ecological perspective (viii) disseminate findings and knowledge gained to all partners and (ix) involve a long-term commitment by all partners. Time was needed to build initially low trust from the community partners in academic researchers and government officials, though community partners were willing to build this trust, and the assets and resources in the community were clearly recognised. It was difficult to agree a common purpose, though, as partnership members had different priorities |
| Kreuter 2012 | Involving representatives from 12 not-for-profit organisations in the program planning facilitated trust building, with intentions to ensure equitable and engaging involvement of community members, including through photovoice. |
| Massi 2023 | Listening and learning from Indigenous community members was paramount, and time was taken to create relationships between the researchers and partner organisations/community members. Providing opportunities and capacity to build skills facilitated involvement from the partner organisation. |
| Rideout 2013 | The community advisory board being responsible for the sessions ensured that initial research areas reflected community concerns. The participatory methods helped to build the relationship between the community advisory board and the steering committee, including because individuals had the option to either speak or write down their ideas. |
| Rikkers 2015 | Conducting telephone interviews helped to save cost and reduce burden on participants. However, there was very low uptake from the telephone survey to attending the community conversations. The participants were not representative of the general Western Australia population, which may reduce transferability. |

**Supplementary Material 6 – Preferred Reporting Items for Systematic reviews and Meta-Analyses extension for Scoping Reviews (PRISMA-ScR) Checklist**

| **Section** | **Item** | **PRISMA-ScR Checklist Item** | **Reported on Page** |
| --- | --- | --- | --- |
| TITLE | | | |
| Title | 1 | Identify the report as a scoping review. | 1 |
| ABSTRACT | | | |
| Structured Summary | 2 | Provide a structured summary that includes (as  applicable): background, objectives, eligibility criteria, sources of evidence, charting methods, results, and conclusions that relate to the review questions and objectives. | 2 |
| INTRODUCTION | | | |
| Rationale | 3 | Describe the rationale for the review in the context of what is already known. Explain why the review questions/objectives lend themselves to a scoping review approach. | 3 |
| Objectives | 4 | Provide an explicit statement of the questions and objectives being addressed with reference to their key elements (e.g., population or participants, concepts, and context) or other relevant key elements used to conceptualize the review questions and/or objectives. | 4 |
| METHODS | | | |
| Protocol and registration | 5 | Indicate whether a review protocol exists; state if and where it can be accessed (e.g., a Web address); and if available, provide registration information, including the registration number. | N/A |
| Eligibility criteria | 6 | Specify characteristics of the sources of evidence used as eligibility criteria (e.g., years considered, language, and publication status), and provide a rationale. | 4 – 5 |
| Information Sources | 7 | Describe all information sources in the search (e.g., databases with dates of coverage and contact with authors to identify additional sources), as well as the date the most recent search was executed. | 4 |
| Search | 8 | Present the full electronic search strategy for at least 1 database, including any limits used, such that it could be repeated. | Supplementary material 1 |
| Selection of sources of evidence | 9 | State the process for selecting sources of evidence (i.e., screening and eligibility) included in the scoping review. | 5 |
| Data charting process | 10 | Describe the methods of charting data from the included sources of evidence (e.g., calibrated forms or forms that have been tested by the team before their use, and  whether data charting was done independently or in duplicate) and any processes for obtaining and confirming data from investigators. | 5, Supplementary Material 2 |
| Data items | 11 | List and define all variables for which data were sought and any assumptions and simplifications made. | 5, Supplementary material 3 |
| Critical appraisal of individual sources of evidence | 12 | If done, provide a rationale for conducting a critical appraisal of included sources of evidence; describe the methods used and how this information was used in any data synthesis (if appropriate). | N/A |
| Synthesis of results | 13 | Describe the methods of handling and summarizing the data that were charted. | 5 |
| RESULTS | | | |
| Selection of sources of evidence | 14 | Give numbers of sources of evidence screened, assessed for eligibility, and included in the review, with reasons for exclusions at each stage, ideally using a flow diagram | 6 |
| Characteristics of  sources of  evidence | 15 | For each source of evidence, present characteristics for which data were charted and provide the citations. | 6 – 10 |
| Critical appraisal  within sources of  evidence | 16 | If done, present data on critical appraisal of included sources of evidence (see item 12). | N/A |
| Results of  individual sources  of evidence | 17 | For each included source of evidence, present the relevant data that were charted that relate to the review questions and objectives. | 6 - 10 |
| Synthesis of results | 18 | Summarize and/or present the charting results as they relate to the review questions and objectives. | 6 – 10 |
| DISCUSSION | | | |
| Summary of evidence | 19 | Summarize the main results (including an overview of concepts, themes, and types of evidence available), link to the review questions and objectives, and consider the  relevance to key groups. | 10 - 12 |
| Limitations | 20 | Discuss the limitations of the scoping review process | 12 |
| Conclusions | 21 | Provide a general interpretation of the results with respect to the review questions and objectives, as well as potential implications and/or next steps. | 12 |
| FUNDING | | | |
| Funding | 22 | Describe sources of funding for the included sources of evidence, as well as sources of funding for the scoping review. Describe the role of the funders of the scoping review. | 13 |
